# Simultaneous fabrication of line and dot dual nanopatterns using miktoarm block copolymer with photocleavable linker

**DOI:** 10.1038/s41467-017-02019-9

**Published:** 2017-11-24

**Authors:** Chungryong Choi, Jichoel Park, Kanniyambatti L. Vincent Joseph, Jaeyong Lee, Seonghyeon Ahn, Jongheon Kwak, Kyu Seong Lee, Jin Kon Kim

**Affiliations:** National Creative Research Center for Block Copolymer Self-Assembly, Department of Chemical Engineering, Pohang University of Science and Technology, Kyungbuk, 790-784 Korea

## Abstract

Block copolymers with various nanodomains, such as spheres, cylinders, and lamellae, have received attention for their applicability to nanolithography. However, those microdomains are determined by the volume fraction of one block. Meanwhile, nanopatterns with multiple shapes are required for the next-generation nanolithography. Although various methods have been reported to achieve dual nanopatterns, all the methods need sophisticated processes using E-beam. Here, we synthesized a miktoarm block copolymer capable of cleavage of one block by ultraviolet. Original cylindrical nanodomains of synthesized block copolymer were successfully transformed to lamellar nanodomains due to the change of molecular architecture by ultraviolet. We fabricated dual nanopatterns consisting of dots and lines at desired regions on a single substrate. We also prepared dual nanopatterns utilizing another phase transformation from spheres to cylinders in a block copolymer with higher interaction parameter. Since our concept has versatility to any block copolymer, it could be employed as next-generation nanolithography.

## Abstract (web summary)

Block copolymers that form nanodomains and are used in nanolithography usually do not form nanopatterns with multiple shapes. Here the authors report a block copolymer that allows for UV- induced cleavage of one arm and demonstrate the transformation of the cylindrical to the lamellar nanodomain.

## Introduction

Block copolymers (BCP) capable of self-assembling to various nanodomains such as lamellae, cylinders, and spheres, have received great attention for next-generation nanolithography, high density storage memory devices, and photonics^1–18^. In particular, next-generation integrated circuits require various shapes and sizes in a single substrate^19–23^. However, BCP self-assembly has a limitation that the shape of nanodomains is only controlled by the volume fraction of one block. Thus, once a single BCP is used, only one shape of nanodomain is obtained^1^.

To overcome this limitation, several approaches have been introduced to fabricate dual nanopatterns on a single substrate. A specially designed substrate was used, where some regions of the substrate were chemically treated, while other regions were not^24, 25^. In this situation, two different orientations of cylindrical nanodomains were achieved: parallel and perpendicularly oriented cylinders. However, the preparation of this kind of patterned substrate needs sophisticated top-down lithography, such as high cost E-beam lithography, which limits applying patterning in a large area. Although some research groups utilized external fields, such as light^26–28^ and electric field^29^ to control the orientation of nanodomains, these approaches have focused on the orientation of a single nanodomain (e.g., cylinders).

To achieve dual nanopatterns in a given substrate, two different nanodomains should be used. The easiest way to pursue this requirement is to use the order-to-order transition (OOT) of a given BCP. Typically, two methods have been widely used to achieve OOT of a BCP: temperature^30–32^ and selective solvent or solvent vapor^33–36^. But, it is not practically possible to make temperature-sensitive patterning in a single substrate, where temperature of one region of the substrate is different from that of other regions. Similarly, one could not easily obtain a substrate that the amount of solvent (or solvent vapor) in one region is different from that at other regions. But, for solvent treatment, one can use crosslinking at desired (or selective) regions in a BCP thin film by irradiation of ultraviolet (UV) or electron beam (E-beam)^37, 38^. The nanodomains of non-crosslinked regions of a BCP are easily changed under solvent vapor annealing, resulting in the formation of different nanodomains from those at crosslinked regions. Recently, Black and coworkers^39^ prepared dual nanopattern (dots and lines) by combination of chemically patterned substrate and the use of the blend of two BCPs having cylindrical and lamellar nanodomains. But, E-beam lithography was needed to fabricate the patterned substrate.

In this study, we introduced a BCP capable of changing its nanodomains by UV irradiation. We synthesized PS(*hv*-PS′)-*b*-PMMA miktoarm BCP by azide-alkyne click reaction^40^. Here, PS and PMMA are polystyrene and poly(methyl methacrylate), respectively, while *hv*-PS′ is another polystyrene having photocleavable linker(*hv*) of *o*-nitrobenzyl alcohol, which is easily cleaved under UV irradiation at 365 nm^41–43^. PS-*b*-PMMA would be one of the strongest candidates for nano lithography because of easy vertical orientation of nanodomains and good dry etching contrast by oxygen reactive ion etching (O_2_ RIE)^44–46^. Also, we synthesized another miktoarm PS(*hv*-PS′)-*b*-poly(2-vinylpyridine) (P2VP) with higher interaction parameter (*χ*) than PS and PMMA^47, 48^. When this BCP is irradiated by UV, the junction point is broken and the resulting BCP becomes the binary blend of PS-*b*-PMMA (or PS-*b*-P2VP) diBCP and PS′ homopolymer. Since the nanodomains of A(*hv*-A′)-*b*-B miktoarm BCP would be different from those of A-*b*-B diBCP (or blend of A-*b*-B diblock and A′ homopolymer) at a given volume fraction of A because of easy formation of curvature at the interface resulting from molecular architecture of A(*hv*-A′)-*b*-B^49–53^, we successfully obtained two different nanodomains by using UV irradiation. Since our concept to fabricate dual nanopatterns is applied to any BCP, it could be used for next-generation nanolithography.

## Results

### Synthesis and characterization

**Fig. 1:**
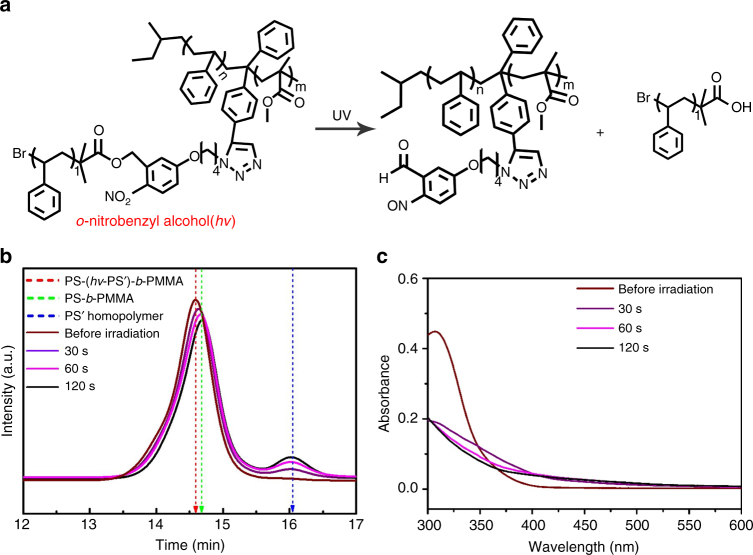
Photocleavage of PS(*hv*-PS′)-*b*-PMMA during UV irradiation at 365 nm. **a** Chemical structure change during photocleavage from PS(*hv*-PS′)-*b*-PMMA to a binary blend of PS-*b*-PMMA and PS′ homopolymer. Changes of **b** SEC trace and **c** UV absorbance of PS(*hv*-PS′)-*b*-PMMA during UV irradiation up to 120 s

PS(*hv*-PS′)-*b*-PMMA was synthesized by azide-alkyne click reaction^40^ of PS-*b*-PMMA with alkyne moiety between the junction point (PS-Alkyne-*b*-PMMA) and homo PS′ containing photocleavable moiety and azide group (PS′-*hv*-N_3_). The chemical structure of PS(*hv*-PS′)-*b*-PMMA is given in Fig. 1a and a detailed synthesis is described in the Supplementary Methods and Supplementary Figs. 1–9. The number average molecular weights of PS, PMMA, and PS′ blocks in PS(*hv*-PS′)-*b*-PMMA were 24,000, 22,000, and 11,000 g mol^−1^, respectively (Supplementary Table 1). All polymers showed narrow polydispersity index of <1.18 (Supplementary Fig. 9A).

We checked whether synthesized PS(*hv*-PS′)-*b*-PMMA was perfectly cleaved to PS-*b*-PMMA diBCP and PS′ homopolymer by UV irradiation with a wavelength of 365 nm. Although PMMA block was degraded under UV irradiation at 254 nm^7, 54^, we found that PMMA chains did not degrade under UV irradiation at 365 nm, confirmed by Fourier transform infrared (FT-IR) spectra, as shown in Supplementary Fig. 10. Figure 1b shows size exclusion chromatography (SEC) traces at various UV irradiation time up to 120 s at 230 °C. The peaks at 14.55 min, 14.70 min, and 16.01 min correspond to PS(*hv*-PS′)-*b*-PMMA, PS-*b*-PMMA, and PS′ homopolymer, respectively. At 30 s irradiation, only a small amount of PS(*hv*-PS′)-*b*-PMMA was cleaved. At 60 s, a peak corresponding to PS′ homopolymer was clearly seen, and the main peak was shifted. Finally, after 120 s irradiation, all of PS(*hv*-PS′)-*b*-PMMA completely split into PS-*b*-PMMA and PS′ homopolymer. The SEC curve obtained after longer irradiation time (say, 180 s) was essentially the same as that at 120 s irradiation (Supplementary Fig. 11). The cleavage during UV irradiation was also confirmed by change of UV absorbance, as shown in Fig. 1c. Before UV irradiation, there was a strong absorbance around 320 nm arising from *o*-nitrobenzyl alcohol moiety. As the irradiation time increased, this absorbance decreased gradually and finally disappeared after 120 s.

### Morphology before and after UV irradiation in bulk state

**Fig. 2:**
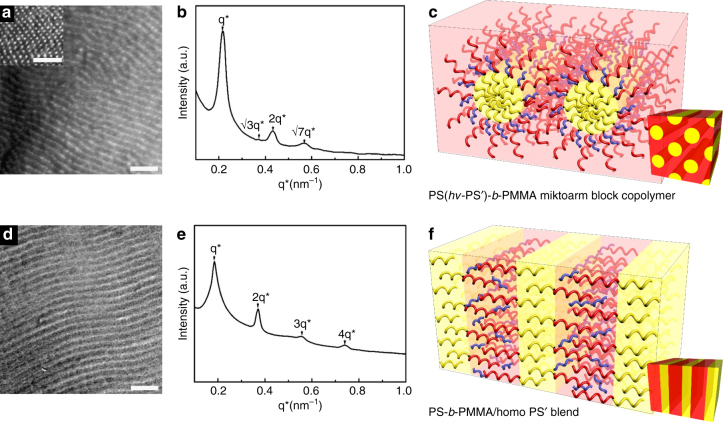
Bulk morphologies before and after UV irradiation. TEM images of PS(*hv*-PS′)-*b*-PMMA **a** before and **d** after UV irradiation. Scale bar is 100 nm. SAXS profiles **b** before and **e** after UV irradiation for 2 h. Schematic of phase transition from cylinders to lamellae **c** before and **f** after UV irradiation

Figure 2 shows transmission electron microscopy (TEM) images and small-angle X-ray scattering (SAXS) profiles (*I*(**q**) vs. **q** ( = (4*π* /*λ*) sin *θ*, where **q** and 2*θ* are the scattering vector and scattering angle, respectively) of PS(*hv*-PS′)-*b*-PMMA before and after UV irradiation. PS nanodomains look dark in TEM images due to selective staining by RuO_4_. PS-Alkyne-*b*-PMMA diBCP showed lamellar nanodomains (Supplementary Fig. 12). But PS(*hv*-PS′)-*b*-PMMA miktoarm BCP clearly showed hexagonally packed cylindrical nanodomains (Fig. 2a). The dot pattern in the inset was observed for the sample cut perpendicularly to the cylinder axis. The hexagonally packed cylinders were also confirmed by the SAXS profile, showing the peaks at q*:$\surd 3$q*:2q*:$\surd 7$q* (Fig. 2b). Generally, a linear A-*b*-B diBCP with a volume fraction of one block having 0.64 shows lamellar nanodomains^55^, while miktoarm BCPs with this volume fraction give cylindrical nanodomains due to the curvature effect in the interface^51^.

When the sample was exposed to UV, the morphology changed to lamellar nanodomains, as demonstrated by TEM image (Fig. 2d), as well as SAXS profile showing the peaks at q*: 2q*: 3q*: 4q* (Fig. 2e). After UV irradiation, PS(*hv*-PS′)-*b*-PMMA becomes a binary blend of PS-*b*-PMMA diBCP and PS′ homopolymer. Since *M*_n_ of PS′ homopolymer is smaller than *M*_n_ of PS-*b*-PMMA diBCP and the volume fraction of PS′ homopolymer in the blend is 0.2, PS′ homopolymer chains are located inside the PS nanodomains, which results in the maintenance of lamellar nanodomains, as shown in Fig. 2f^56, 57^. Since PS′ homopolymer is located in the PS lamellar nanodomains, the domain spacing (33.9 nm) of the binary blend is slightly increased compared with that (31.3 nm) of neat PS-Alkyne-*b*-PMMA (Supplementary Fig. 12). This is consistent with the experimental result of binary blend of a diBCP and a homopolymer^58^. The first peak position (q*) during UV irradiation was changed from 0.2179 to 0.1851 nm^−1^, indicating that the domain spacing (*L*_o_) was increased from 28.8 to 33.9 nm. The increased domain spacing was attributed to the nanodomain transformation from cylinders to lamellae^59^.

### Dot and line dual nanopatterns in thin film

We investigated thin film morphology for applying to nanolithography, where vertically oriented cylindrical and lamellar nanopatterns are needed. We used a silicon substrate treated by PS-*r*-PMMA copolymer brush for the surface neutralization. PS(*hv*-PS′)-*b*-PMMA was spin coated on a neutralized silicon wafer with a thickness 57 nm(~2*L*_0_).

**Fig. 3:**
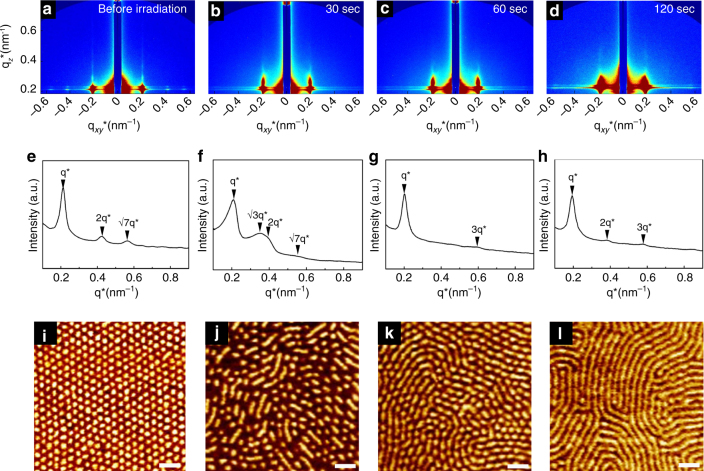
Thin film morphologies during UV irradiation at 230 °C. **a**–**d** GISAXS patterns, **e**–**h** the in plane (**q**_*xy*_) scans of GISAXS patterns, and **i**–**l** phase contrast AFM images at various UV irradiation times. **a, e, i**: before irradiation; **b, f, j**: 30 s; **c, g, k**: 60s; **d, h, l**: 120s. The thickness of the sample is 57 nm (~2*L*_0_). Scale bar is 100 nm

Figure 3 gives graze-incidence SAXS (GISAXS) images and in-plane scattering profiles, as well as phase contrast atomic force microscopy (AFM) images of the thin films at various UV exposure times at 230 °C. Before the irradiation, the samples clearly showed hexagonally packed cylinders (Fig. 3a, e) because of clear peaks observed at 2q* and $\surd 7$q*. Also, all cylindrical nanodomains are vertically oriented to the substrate due to the streaks in GISAXS patterns along the **q**_*z*_ direction (out of plane and normal to the film surface) (Fig. 3a). Furthermore, AFM images demonstrate the vertically oriented cylinders. The vertical orientation of cylinder nanodomains is attributed to the neutral surface. At 30 s, although the vertical orientation is maintained, the SAXS profiles show the coexistence of cylinders and lamellae, which are also clearly demonstrated by AFM image (Fig. 3j). Finally, vertically oriented lamellar nanodomains are obtained at 120 s irradiation because of clear SAXS peaks at q*, 2q*, and 3q*, as well as the streaks along the **q**_*z*_ direction. Also, AFM image shows the vertically oriented lamellae. From Fig. 3e, h, the domain spacing (2$\pi$/q*) of lamellar nanodomains is larger than that of cylindrical nanodomains, consistent with bulk morphology transition, as shown in Fig. 2b, e. These results are completely consistent with SEC traces and UV absorbance (Fig. 1b, c). At 30 s, because some parts of PS(*hv*-PS′)-*b*-PMMA split into PS-*b*-PMMA and PS′ homopolymer, cylindrical and lamellar nanodomains coexist. At 60 s, most parts are cleaved and connected cylinders are observed. Finally, when all PS(*hv*-PS′)-*b*-PMMA completely split into a binary blend of PS-*b*-PMMA and PS′ homopolymer at 120 s irradiation, entirely vertically oriented lamellae are observed.

We investigated the effect of film thickness and UV irradiation temperature on the phase transformation. For a smaller film thickness (28 nm (~1*L*_0_)), we also obtained vertically oriented lamellae transformed from vertically oriented cylinders by UV irradiation (Supplementary Fig. 13). On the other hand, the sample was exposed at lower temperatures (for instance, 180 °C), much longer time (~2 h) was needed to complete photocleavage (Supplementary Fig. 14) and the phase transformation (Supplementary Fig. 15).

**Fig. 4:**
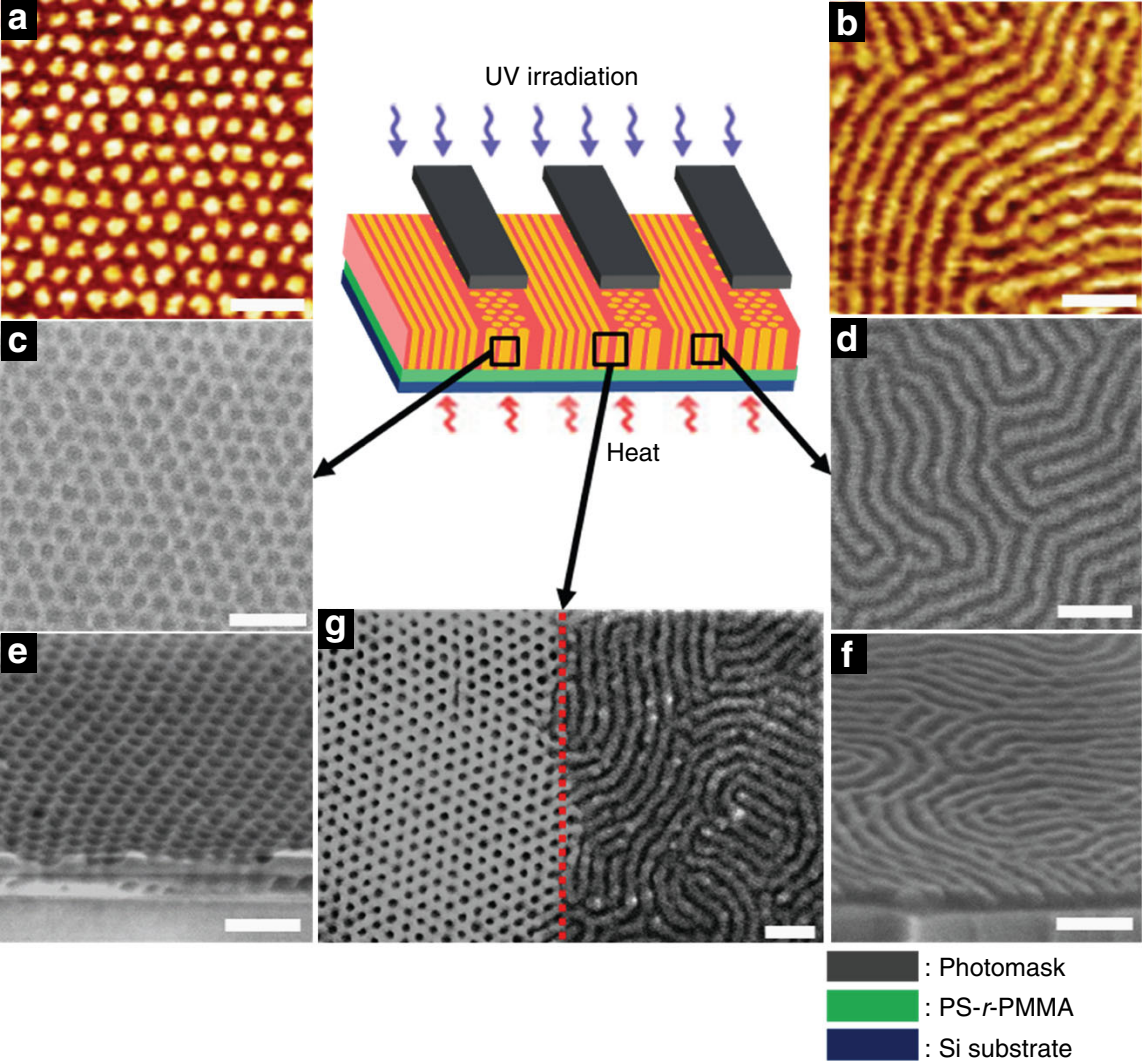
Thin film morphology after selective UV exposure. **a**, **c**,** e** Vertically oriented cylindrical nanodomains at unexposed regions and **b, d,** **f** vertically oriented lamellar nanodomain at UV exposed regions. **a**,** b** Phase contrast AFM images. **c**,** d** Plane view and **e**,** f** cross-sectional SEM images. **g** SEM image of the boundary between unexposed and UV exposed regions. All of SEM images are obtained after selective removal of PMMA using O_2_ RIE. The thickness of the sample is 57 nm (~2*L*_0_). Scale bar is 100 nm

Since we used UV irradiation, we could fabricate dual nanopatterns at the desired areas in a single substrate by using a photomask. UV irradiation was blocked at the area below the photomask. Figure 4a, b show that vertically oriented cylinders remained at unexposed (UV-blocked) areas, while the exposed regions exhibited vertically oriented lamellae. Figure 4c–f give top and cross-sectional scanning electron microscopy (SEM) images after selective removal of PMMA block by O_2_ RIE. Vertically aligned lamellae and cylinders are spanned through the entire film thickness. We also observed the boundary between exposed and unexposed areas by SEM, as given in Fig. 4g. It is clear to demonstrate the transition between vertically aligned cylinders and lamellae at this boundary. This indicates that when one uses a more sophisticated photomask, the boundary between two patterns would be much sharper.

**Fig. 5:**
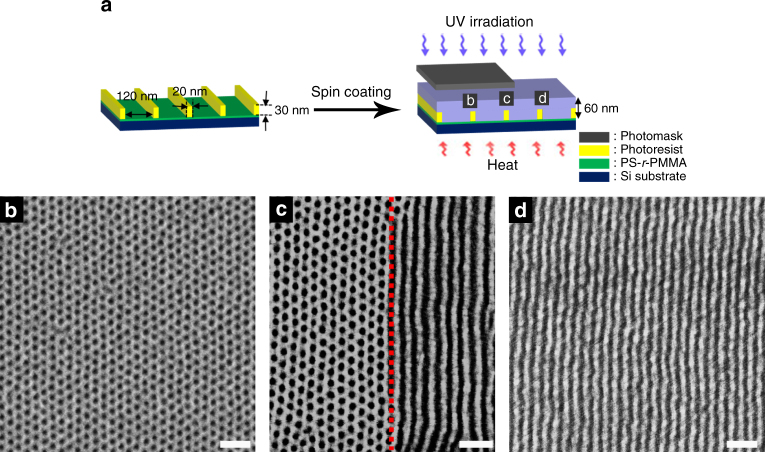
Directed self-assembly of PS(*hv*-PS′)-*b*-PMMA after selective UV exposure. **a** Scheme of directed self-assembly using topologically pre-patterned substrate. SEM images of PS(*hv*-PS′)-*b*-PMMA on a patterned substrate at **b** unexposed, **d** exposed, and **c** boundary regions. Dark holes and lines in SEM images represent PMMA nanodomains that were etched out by O_2_ RIE. The red dotted line represents the boundary. Scale bar is 100 nm

For industrial purposes, directed self-assembly (DSA)^6, 9^ is needed to obtain long-range lateral ordering. For this purpose, we used a topologically pre-patterned substrate^60–62^ and the BCP film thickness was ~60 nm, as shown in Fig. 5a. Figure 5b–d show a good lateral long-range ordering of vertically aligned cylinders and lamellae at unexposed and exposed regions, respectively. Also, we clearly demonstrated a sharp boundary between vertically oriented cylinders and lamellae (Fig. 5c). Thus, we successfully fabricated well aligned dots and lines dual nanopatterns at selective (or desired) regions in a single substrate.

Finally, to verify the versatility of our concept to fabricate dual nanopatterns based on miktoarm BCPs with a photocleavable linker, we chose another phase transition from spheres to cylinders. We synthesized PS(*hv*-PS′)-*b*-P2VP. *χ* of PS and P2VP blocks is much larger than that of PS and PMMA^47^, which would also an advantage to obtain sub 10 nm pattern for the next-generation lithography^48^. The detailed synthesis and characterization are described Supplementary Fig. 16. The molecular weight of PS, P2VP and PS′ blocks of PS(*hv*-PS′)-*b*-P2VP are 32,500, 12,500, and 15,000 g mol^−1^, respectively, and the volume fraction of total PS in the BCP (PS + PS′) is 0.80. Pure PS(*hv*-PS′)-*b*-P2VP showed body-centered cubic spherical microdomains, as expected for the volume fraction of PS of 0.8. After the UV irradiation, the mixture of PS-(*hv*-PS′)-*b*-P2VP and homo PS showed hexagonally packed cylindrical microdomains (Supplementary Fig. 17). By using thin film, we successfully fabricated dual nanopatterns consisting of dots and lines resulting from spherical and parallel oriented cylindrical nanodomains, respectively, at the desired areas. In addition, we observed, via AFM, a sharp boundary between spherical and cylindrical nanopatterns (Supplementary Fig. 18). Because a metal precursor is selectively incorporated into P2VP nanodomains^63, 64^, we could fabricate, via DSA, a long-range ordering of dual nanopatterns consisting of platinum nanodots and nanowires at the desired areas, as shown in Supplementary Fig. 19. It is noted that metal nanostructures have potential applications such as electronics, photonics, and catalyst^65–67^.

In conclusion, we have designed and synthesized a PS(*hv*-PS′)-*b*-PMMA miktoarm BCP with UV-cleavable moiety, which becomes a binary blend of PS-*b*-PMMA diBCP and PS′ homopolymer by UV irradiation. By judicious control of molecular weights of each arm in the miktoarm BCP, we achieved a transformation of the original cylindrical nanodomains to lamellar nanodomains by UV irradiation.

Since UV irradiated area and position are easily tuned using a photomask, we could fabricate dual nanopatterns at the desired areas. Namely, some regions have vertically oriented lamellae (or line nanopatterns), while other regions exhibit vertically oriented cylinders (or dot nanopatterns). Finally, by combination of directed self-assembly and UV irradiation, well aligned dots and lines dual nanopatterns were fabricated at specific (or desired) regions in a single substrate. We also demonstrated that dual nanopatterns were fabricated by using PS(*hv*-PS′)-*b*-P2VP with higher *χ*. Dual nanopatterns fabricated in this study could be used for next-generation nanolithography. Although a silicon-containing BCP (for instance, PS-*b*-poly(dimethyl siloxane) with higher *χ* and excellent etching selectivity between blocks) with photocleavable unit could be a very promising for the fabrication of dual nanopattern, it would be an interesting future work.

## Methods

### Synthesis of miktoarm block copolymer

The detailed synthetic method is described in the Supplementary Methods. Before synthesizing polymers, we prepared atom-transfer radical polymerization (ATRP) initiator containing photocleavable moiety, azide group, and functionalized diphenyl ethylene with alkyne. Using ATRP initiator, PS-*hv*-N_3_ was polymerized at 90 °C in oil bath in the presence of purified copper(I) bromide (CuBr), N,N,N′,N″,N″-pentamethyldiethylenetriamine (PMDETA) as catalyst. The molecular weight was controlled by reaction time. Then, we synthesized PS-Alkyne-*b*-PMMA protected with (*tert*-butyldimethylsilyl)acetylene (TBDMS) by anionic polymerization at −78 °C in tetrahydrofuran under argon environment. Deprotection of TBDMS was done by putting tetrabutylammonium fluoride solution in THF solution for 12 h at room temperature under argon environment. Finally, we used click reaction between PS-*hv*-N_3_ and PS-Alkyne-*b*-PMMA in the presence of CuBr, PMDETA in THF for 2 days at room temperature^40^. The reacted solution was passed through alumina column to remove copper catalyst and dropped in cyclohexane to remove unreacted PS-*hv*-N_3_.

### Characterization of PS(*hv*-PS′)-*b*-PMMA block copolymer

For cleavage by UV irradiation, we made PS(*hv*-PS′)-*b*-PMMA film with a thickness of 3 μm by casting of THF solution on a glass. The film was placed under UV (Photo electronics SPOT LED 15, 365 nm) irradiation for various times up to 120 s under vacuum at 230 °C. Exposed samples were dissolved in THF and analyzed by GPC (Waters 2414 refractive index detector) with two 300 (length) × 7.5 mm (inner diameter) columns including particle size of 5 μm (PLgel 5 μm MIXED-C: Polymer Laboratories) with THF as an eluent and a flow rate of 1 mL min^−1^ at 30 °C. Also, we obtained UV absorbance (Agilent Cary 5000) of the film irradiated by UV for various times.

### Bulk morphologies

We prepared two films on a Teflon sheet. One was exposed to UV (365 nm) for 2 h at 230 °C, while the other was not. Then, both samples were further annealed at 230 °C for 3 days under vacuum, and quenched to room temperature. To prepare TEM samples, the samples were sectioned by ultramicrotome (Leica EM UC6) with 40 nm thickness and transferred to a copper grid, and strained by RuO_4_ for 5 min to selectively stain PS block. The morphologies were observed by TEM (Hitachi 7600). SAXS measurements were conducted at room temperature on 4 C beamline at Pohang Accelerator Laboratory (PAL, South Korea), where the X-ray wavelength was 0.1608 nm. The sample to detector distance was 4 m and the scattered X-rays were collected on a 2D CCD detector (Princeton Instruments, SCX-TE/CCD-1242).

### Thin film morphologies

PS-*r*-PMMA (mole fraction of PS = 57% and *M*_n_ = 6500 g mol^−1^ purchased by Polymer Source) in toluene (1.0 wt%) was spin coated on silicon wafer cleaned by piranha solution (sulfuric acid: hydrogen peroxide = 3:1) followed by drying with nitrogen gas. The film was placed in vacuum oven at 180 °C for three days. After thermal treatment, the residual random copolymer was rinsed by toluene. Two different film thicknesses of PS(*hv*-PS′)-*b*-PMMA with 28 nm (~1*L*_0_) and 57 nm (~ 2*L*_0_) were prepared by spin-coating on a silicon wafer using different concentrations of 0.9 wt% and 1.5 wt% in toluene, respectively, at an rpm of 3000. Some areas of the thin film were covered with a chrome mask to screen UV light and put it into a vacuum oven at 230 °C for 120 s under UV irradiation at 365 nm and the sample was annealed for 2 h at 230 °C. Then, the sample was quenched at room temperature. Surface morphologies were observed by AFM (Veeco DI dimension 3100 with Nanoscope V) in the tapping mode. For SEM (Hitachi S4800 working at 3 kV) image, the thin film was etched by O_2_ RIE (30 W and 100 sccm for 45 s) to completely remove PMMA block.

To fabricate topographic patterned substrate for DSA, PS-*r*-PMMA chains were first grafted onto a silicon wafer for surface neutralization and ungrafted PS-*r*-PMMA was removed by toluene. A positive photoresist (PMMA, *M*w = 950,000 g mol^−1^) was spin coated on the neutral brush layer and baked at 160 °C for 1 min to remove the residual solvent of anisole. Then, it was exposed to ArF with a wavelength of 193 nm by using a pre-patterned mask. Finally, the exposed region of the photoresist was removed by propylene glycol methyl ether acetate for 40 s and dried at 100 °C for 1 min. The resulting rectangular shaped trench has a width of 120 nm and a depth of 30 nm. The width of the mesa (the distance between two neighboring trenches) is 20 nm.

GISAXS experiments were performed at room temperature on beam line 3 C at PAL. The incidence angle was 0.16^o^ and the wavelength was 0.1278 nm. Sample to detector distance was set at 4 m.

### Data availability

All data are available from the authors upon reasonable request.

## Electronic supplementary material

Supplementary Information
