# Supplementary material for: Simultaneous fabrication of line and dot dual nanopatterns using miktoarm block copolymer with photocleavable linker
Source: Nat Commun. 2017 Nov 24;8:1765. doi: 10.1038/s41467-017-02019-9 (PMC5701260; doi:10.1038/s41467-017-02019-9)
Supplement: Supplementary file 1 — Supplementary Information [file 41467_2017_2019_MOESM1_ESM.pdf]

Supplementary Methods

PS(*hv*-PS')-*b*-PMMA miktoarm block copolymer was synthesized by azide-alkyne click reaction, as shown in Supplementary Figure 1. All chemicals were purchased from Sigma-Aldrich and used without purification except methyl methacrylate (MMA), styrene, piperidine, tetrahydrofuran (THF) and Copper(I) bromide (CuBr). MMA and styrene were distilled before use to remove inhibitor. For further purification, MMA and styrene were stirred with trioctyl aluminum and di-*n*-butylmagnesium, respectively. Piperidine was also distilled for purification. THF used for anionic polymerization was distilled in sodium/benzophenone mixture and stirred until the color was changed to violet, meaning oxygen free. CuBr was purified by washing with acetic acid, ethanol and diethyl ether, subsequently.

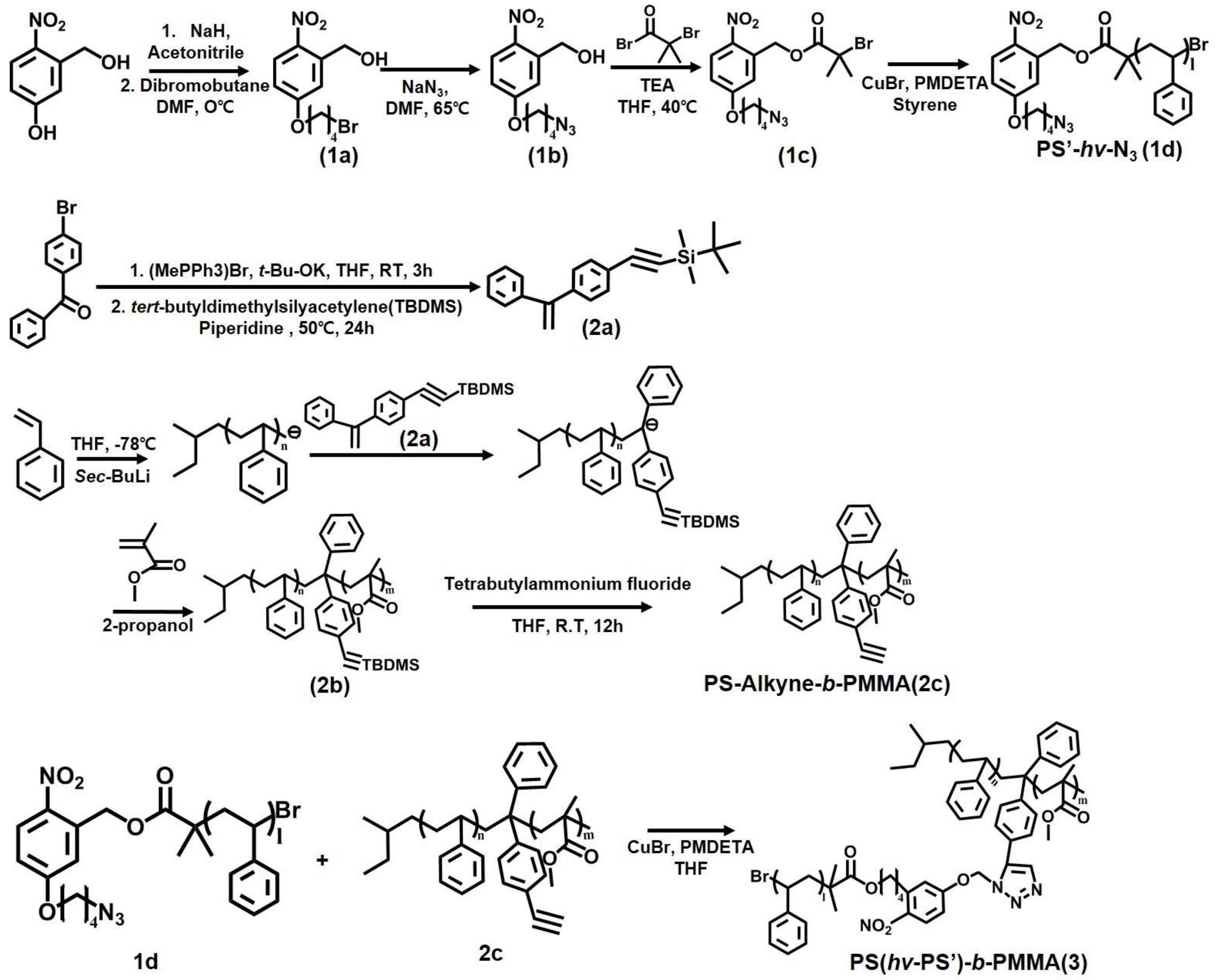

Supplementary Figure 1. Synthetic scheme for PS containing photo-cleavable moiety and azide group (PS'-*hv*-N_3) (**1d**), PS-*b*-PMMA with alkyne (PS-Alkyne-*b*-PMMA) (**2c**) and PS(*hv*-PS')-*b*-PMMA miktoarm block copolymer (**3**).

Synthesis of of PS'-*hv*-N_3 (1d)

Sodium hydride (NaH, 2.16g, 90mmol) aqueous solution was added to 5-hydroxy-1-nitrobenzyl alcohol (5g, 29.6mmol) in acetonitrile solution at 0 °C under nitrogen environment. Yellow precipitation was obtained because 5-hydroxy group was substituted by $-O^- Na^+$. The precipitation was filtered and dissolved in DMF (150ml). Then, dibromobutane (7.03g, 32.56mmol) was added in DMF solution and the solution was stirred for 12 h at room temperature. After reaction, it was quenched by pouring deionized (DI) water and extracted with ethyl acetate. Finally, purified molecule (**1a**) was obtained by conducting column chromatography and it was dried in vacuum oven. Proton nuclear magnetic resonance (^1H NMR) (400 MHz. $CDCl_3$, δ): 8.06(d, 1H), 7.12(s, 1H), 6.78(d, 1H), 4.88(s, 2H), 4.02(t, 2H), 3.32(t, 2H), 3.20(s, 1H), 1.98(m, 2H), 1.90(m, 2H) (Supplementary Fig. 2).

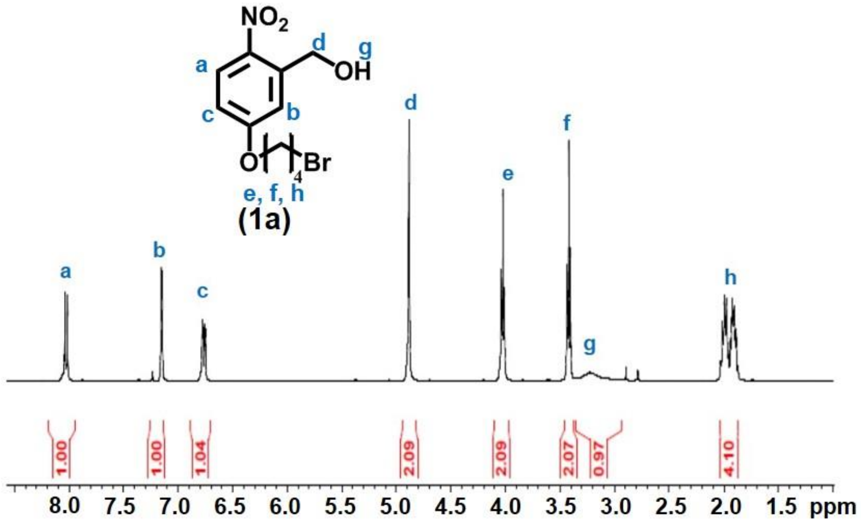

Supplementary Figure 2. ^1H NMR spectra of molecule **1a**.

1a (8.5g, 27.9mmol) and NaN_3 (2.36g, 36.3mmol) were dissolved in acetone:DI water (6:1) mixed solvent and refluxed at 65°C with reflux under nitrogen environment. After column chromatography, purified product (**1b**) was obtained. ^1H NMR (400 MHz. $CDCl_3$, δ): 8.06(d, 1H), 7.12(s, 1H), 6.78(d, 1H), 5.52(s, 2H), 4.02(t, 2H), 3.32(t, 2H), 1.98(m, 2H), 1.90(m, 2H). (Supplementary Fig. 3).

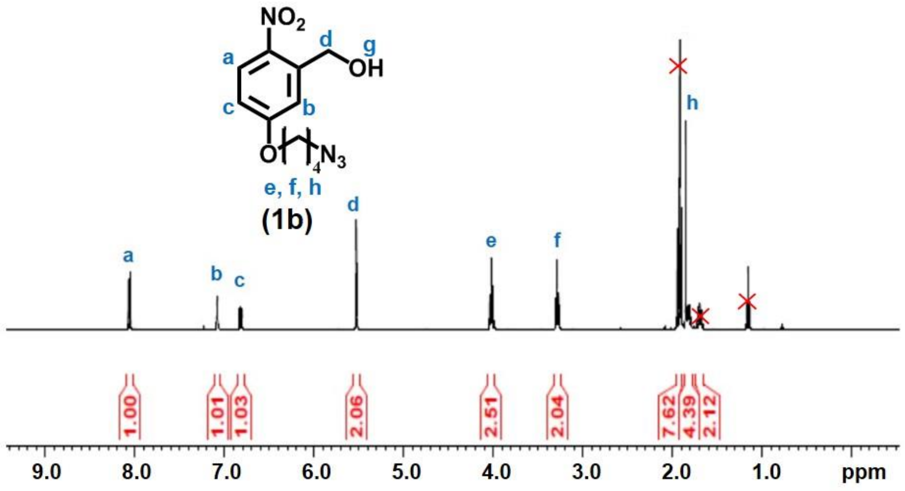

Supplementary Figure 3. ^1H NMR spectra of molecule **1b**.

Triethylamine (3.24g, 32mmol) and 2-bromo-2-methylpropanoyl bromide (7.45g, 32.4mmol) were subsequently added to **1b** (7.19g, 27.0mmol) in THF solution at 40 °C. The salt solution was filtered and the final product (**1c**) was obtained by column chromatography. ^{1}H NMR (400 MHz. $CDCl_3$, δ): 8.06(d, 1H), 7.12(s, 1H), 6.78(d, 1H), 5.52(s, 2H), 4.02(t, 2H), 3.32(t, 2H), 1.99(s, 6H), 1.98(m, 2H), 1.90(m, 2H). Electrospray ionization (ESI) mass spectrum: Mass:415.24 g mol^{-1} (Supplementary Fig. 4).

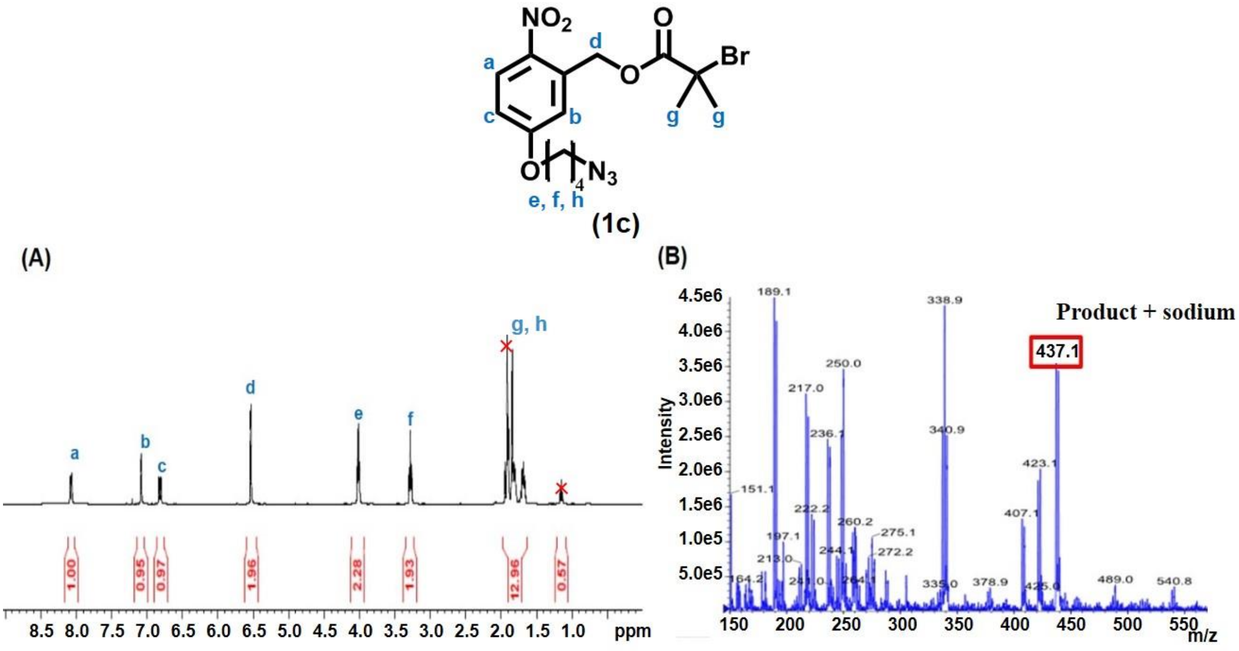

Supplementary Figure 4. (**A**) ^{1}H NMR spectra and (**B**) electrospray ionization mass spectrum of molecule **1c**.

We synthesized PS'-*hv*-N_3 (**1d**) using synthesized ATRP initiator containing photo-cleavable linker and azide group (**1c**). We put the initiator (50 mg), styrene monomer (6 ml), CuBr (18 mg) and N,N,N′,N″,N″-pentamethyldiethylenetriamine (PMDETA, 24μl) into reactor together and conducted freeze-thaw three times and the reactor was stirred in oil bath at 90 °C. We controlled the molecular weight by reaction time. After reaction, the solution passed through an alumina column and was precipitated in methanol to remove copper catalyst. The product, PS'-*hv*-N_3, was characterized by size exclusion chromatography (SEC). Number average molecular weight = 11,000 g mol^{-1} and polydispersity index (PDI) = 1.18 (Supplementary Fig. 5).

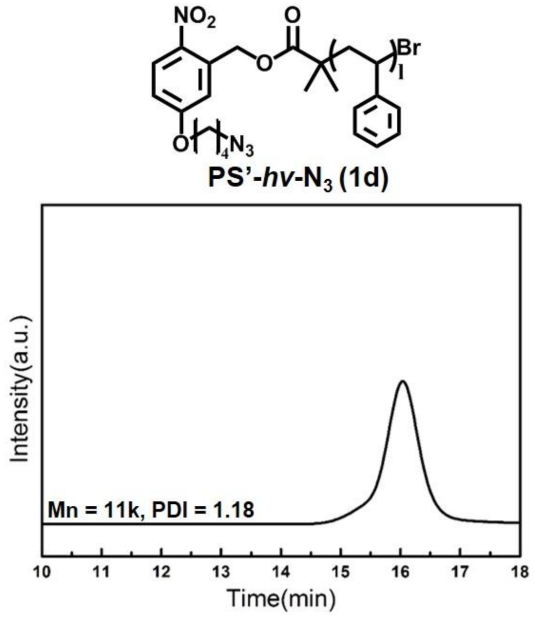

Supplementary Figure 5. SEC trace of PS'-*hv*-N_3 (**1d**).

Synthesis of functionalized diphenyl ethylene (DPE) with alkyne(2a) and polymerization of PS-Alkyne-*b*-PMMA(2c)

We synthesized functionalized diphenyl ethylene (DPE) according to the literature[1, 2]. From *p*-bromobenzophenone, pure functionalized DPE (**2a**) was obtained by Wittig reaction and protection with (*tert*-butyldimethylsilyl) acetylene (TBDMS). ^{1}H NMR (400 MHz. $CDCl_3$, δ): 7.33(d, 2H), 7.19(d, 5H), 7.05(d, 2H), 5.32(d, 2H), 1.03(s, 9H), 0.22(s, 6H) (Supplementary Fig. 6).

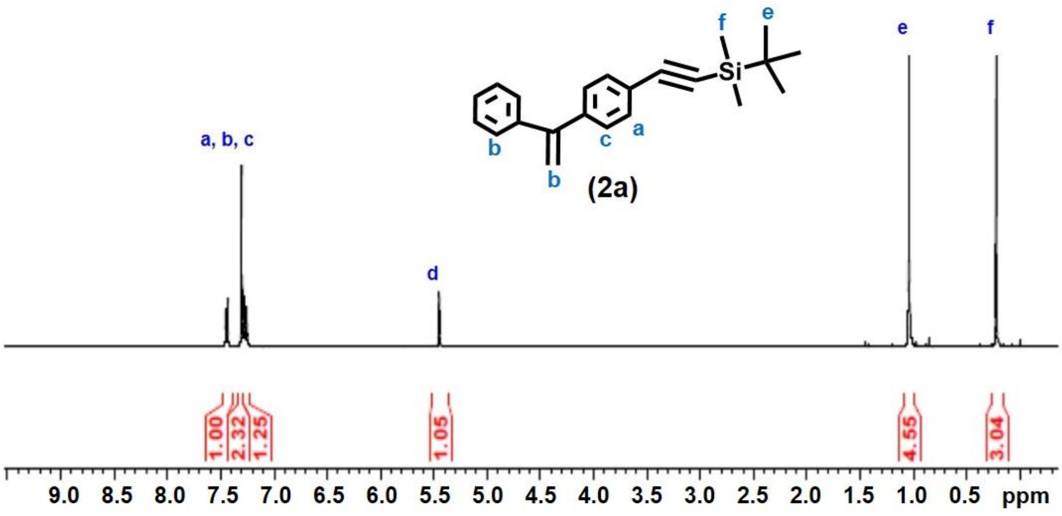

Supplementary Figure 6. ^{1}H NMR spectra of molecule **2a**.

PS-Alkyne-*b*-PMMA with TBDMS protection group (**2b**) was synthesized by anionic polymerization using **2a**. Note that the protecting group of TBDMS is necessary to polymerize PS-Alkyne-*b*-PMMA, because the anion attacks alkyne group during polymerization. *Sec*-butyllithium solution as an initiator (1.2M in cyclohexane, 86μl), styrene monomer (2.48g), **2a** (100 μl, excess), methyl methacrylate (2.27g) and 2-propanol (3ml) as terminator were subsequently added to THF (80ml) in the presence of LiCl (0.3g) at -78°C. The block copolymer (**2b**) was analyzed by GPC and the weight fraction of each block was determined by NMR (Supplementary Fig. 7).

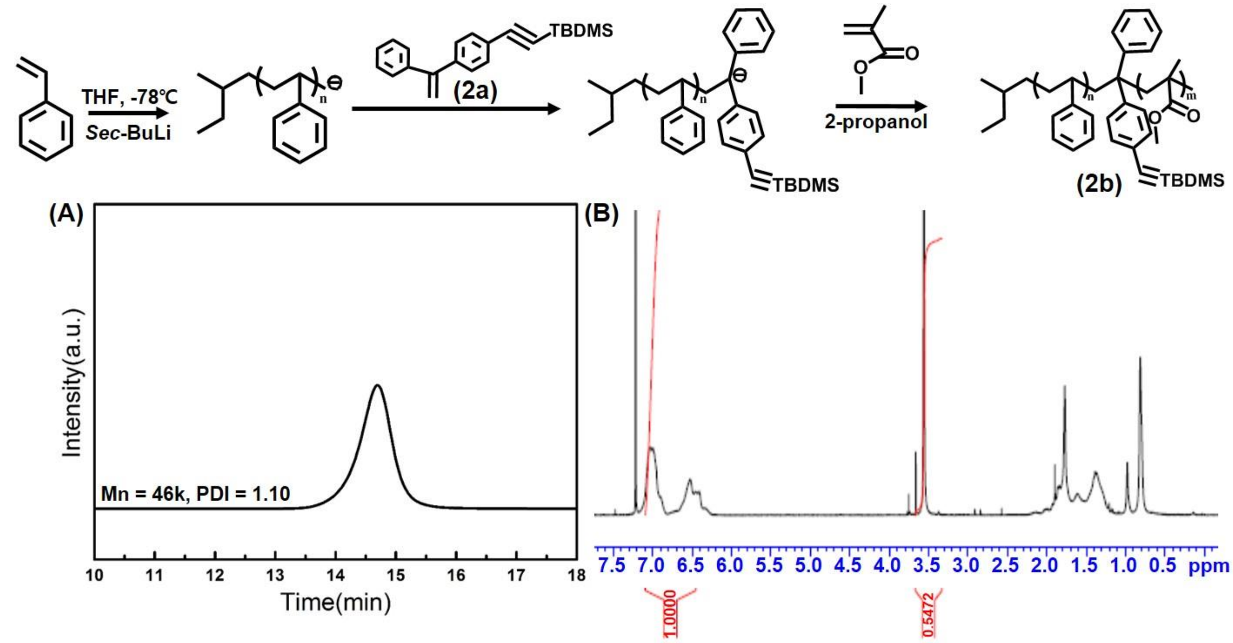

Supplementary Figure 7. (**A**) SEC trace and (**B**) ^{1}H NMR spectra of **2b**.

Deprotection of TBDMS group in **2b** was conducted by adding tetrabutylammonium fluoride solution (TBAF, 1.0M in THF, 10ml). The solution was stirred for 12 h at room temperature. After deprotection, solvent was changed from THF to chloroform, then the solution passed through alumina column for purification. After evaporation of chloroform, deprotected PS-Alkyne-*b*-PMMA (**2c**) was precipitated in methanol. The deprotection was confirmed by ^{1}H NMR. The peak at ~ 0.2 ppm corresponding to the proton near Si atom in TBDMS completely disappeared (Supplementary Fig. 8)[2].

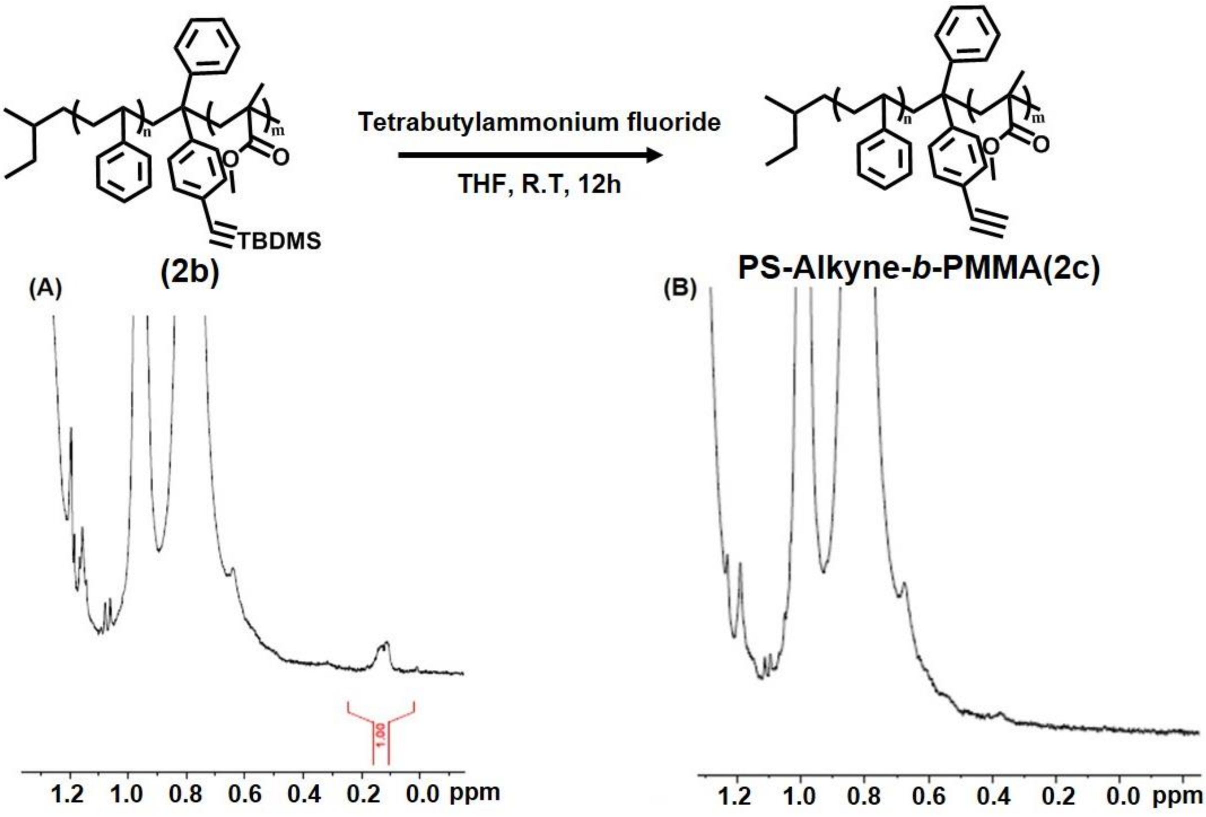

Supplementary Figure 8. ^{1}H NMR spectra of protection group of TBDMS (**A**) before and (**B)** after deprotection.

Coupling reaction between PS'-*hv*-N_3 and PS-Alkyne-*b*-PMMA by click reaction

PS'-*hv*-N_3 (**1d**) (0.0312g, 1.3eq) and PS-Alkyne-*b*-PMMA (**2c**) (0.1g, 1eq) were dissolved in THF (5ml) and the solution was purged with argon. PMDETA (24μl) and CuBr (18mg) were added under argon environment. The solution was stirred for two days at room temperature. After reaction, the solution was passed through an alumina column to remove CuBr catalyst. Since we used an excess amount of PS'-*hv*-N_3, the crude PS(*hv*-PS')-*b*-PMMA contained unreacted PS-*hv*-N_3. When this was removed by precipitating in cyclohexane as a selective solvent for PS, we obtained pure PS(*hv*-PS')-*b*-PMMA (**3**) (Supplementary Fig. 9A). Although the molecular weight of a miktoarm block copolymer would be different from that measured by SEC based on linear PS standards, the molecular weight of synthesized PS(*hv*-PS')-*b*-PMMA is slightly smaller than the sum of two molecular weights (PS-*hv*-N_3 and PS-Alkyne-*b*-PMMA). This is because the molecular weight of PS' homopolymer is smaller than PS-Alkyne-*b*-PMMA; thus hydrodynamic volume of PS(*hv*-PS')-*b*-PMMA miktoarm block copolymer is not much changed compared to linear one at a given molecular weight. The volume fraction of each block was determined by ^{1}H NMR (Supplementary Fig. 9B). Molecular characteristics of polymers synthesized in this study are shown in supplementary Table 1.

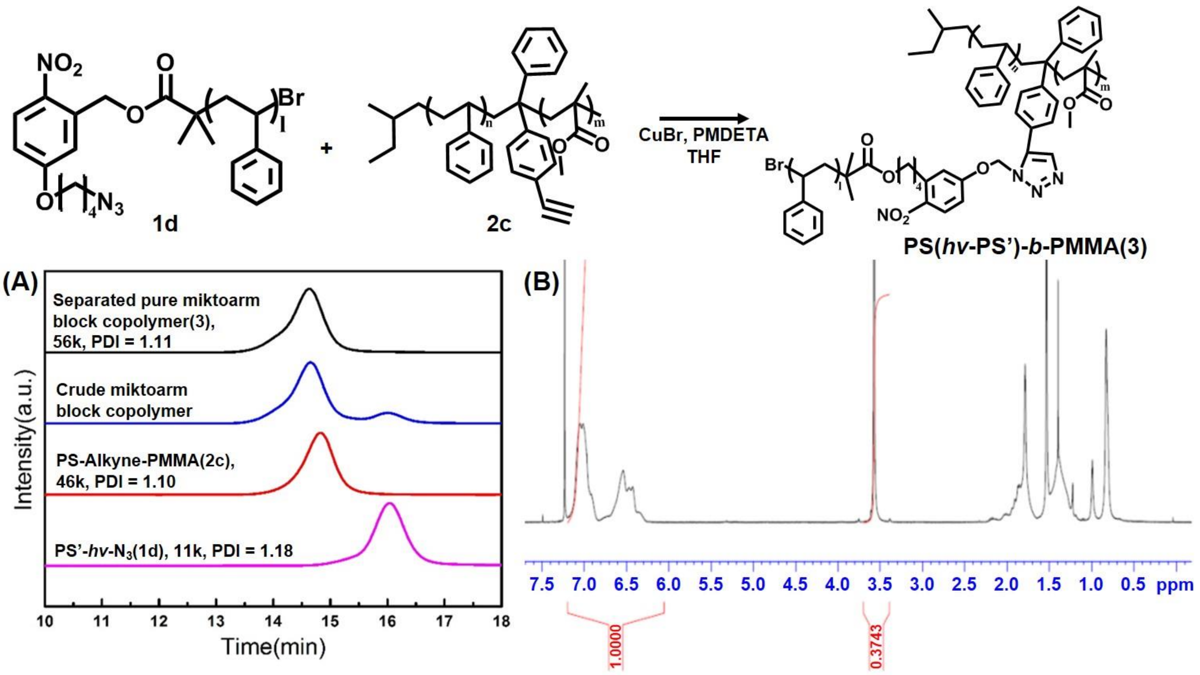

Supplementary Figure 9. Upper Panel: Synthetic scheme to obtain PS-(*hv*-PS')-*b*-PMMA (**3**) by click reaction between PS-*hv*-N_3 (**1d**) and PS-Alkyne-*b*-PMMA (**2c**). Lower Panel: (**A**) SEC trace of PS-*hv*-N_3, PS-Alkyne-*b*-PMMA and PS(*hv*-PS')-*b*-PMMA before and after removal of unreacted PS-*hv*-N_3. (**B**) ^{1}H NMR of pure PS-(*hv*-PS')-*b*-PMMA (**3**).

Supplementary Table 1. Molecular characteristics of PS-Alkyne-*b*-PMMA, PS-*hv*-N_3 and PS(*hv*-PS')-*b*-PMMA

**Polymer**	**$M_{n,Total}$ a)** **(g mol^{-1})**	**$M_{n,PS}$ a)** **(g mol^{-1})**	**$M_{n,PMMA}$ a)** **(g mol^{-1})**	**M_w/M_n a)**	***Vol_{PS}*** **b)**
PS-Alkyne-*b*-PMMA	46,000	24,000	22,000	1.10	0.55
PS'-*hv*-N_3		11,000		1.18	
PS(*hv*-PS')-*b*-PMMA	56,000	35,000	22,000	1.11	0.64

a) M_n and M_w are the number and weight average molecular weights determined based on PS standard. **b)** calculated by ^{1}H NMR using known density of PS (1.05 g cm^{-3}) and PMMA (1.18g cm^{-3}).

Supplementary Figures

FT-IR spectra of PMMA homopolymer before and after UV irradiation

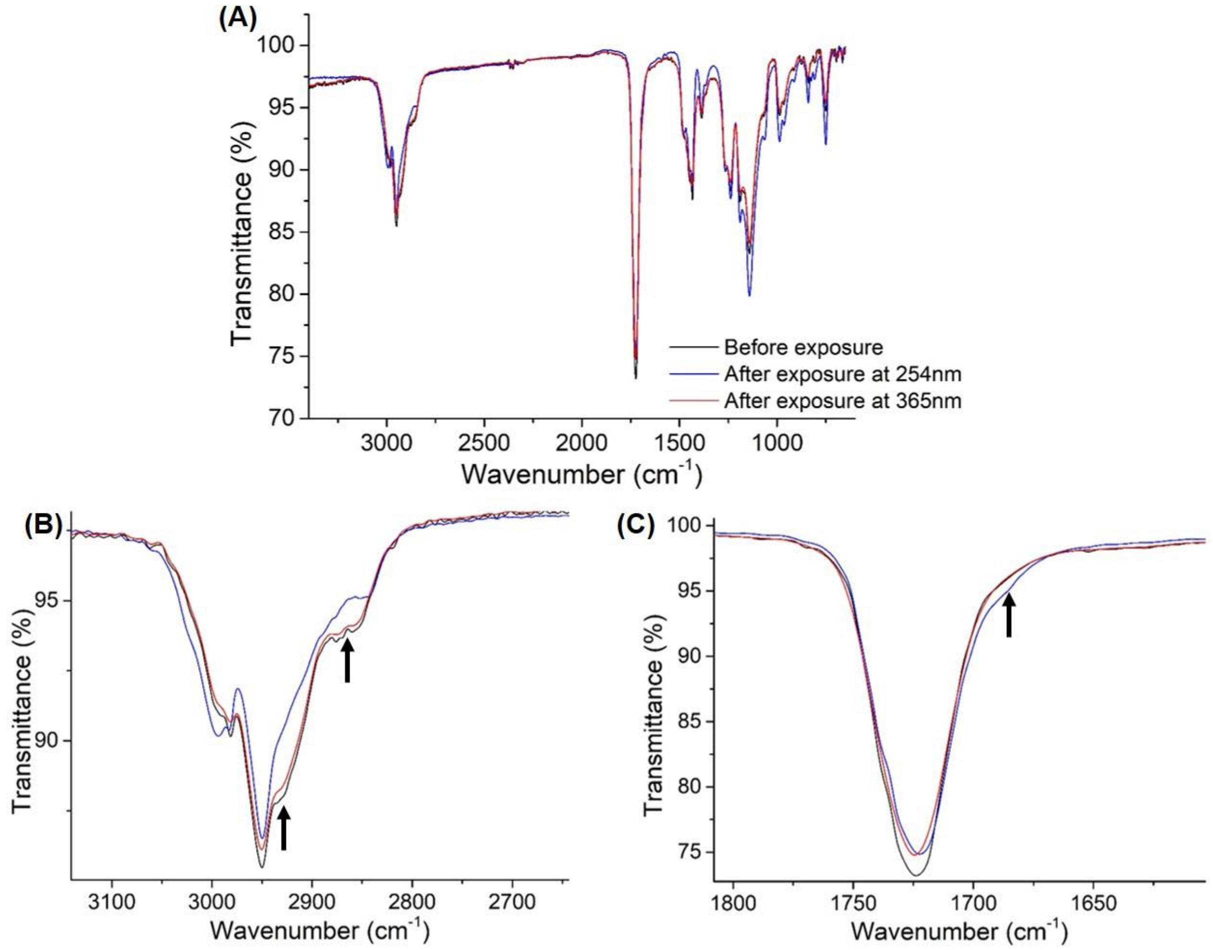

Supplementary Figure 10. (**A**) FT-IR spectra of PMMA homopolymer before and after UV irradiations at two different wavelengths (254 and 365 nm). (**B**) Expanded FT-IR spectra focusing on methyl/methylene stretching band. (**C**) Expanded FT-IR spectra focusing on carbonyl stretching band.

It is well known that PMMA chains are degraded under UV irradiation at 254 nm[3,4]. To investigate any degradation of PMMA chains upon UV irradiation at 365 nm, we obtained FT-IR (JASCO, FT/IR-4600) spectra, as shown in Supplementary Figure 10. When PMMA was exposed by UV irradiation at 254 nm, the intensity of methyl/methylene stretching band at

2840-2935 cm^{-1} decreased compared with neat PMMA, as evidently by two arrows in Supplementary Figure 10B. Also, the carbonyl stretching band showed a shoulder at 1670-1700 cm^{-1}. This clearly indicates the degradation of PMMA chains upon UV irradiation at 254 nm, which is consistent with the result reported in the literature[4]. On the other hand, these peaks after UV irradiation at 365 nm were essentially the same as those before UV irradiation, indicating that no degradation of PMMA chains occurred under UV irradiation at 365 nm.

SEC traces and UV absorbance at longer irradiation

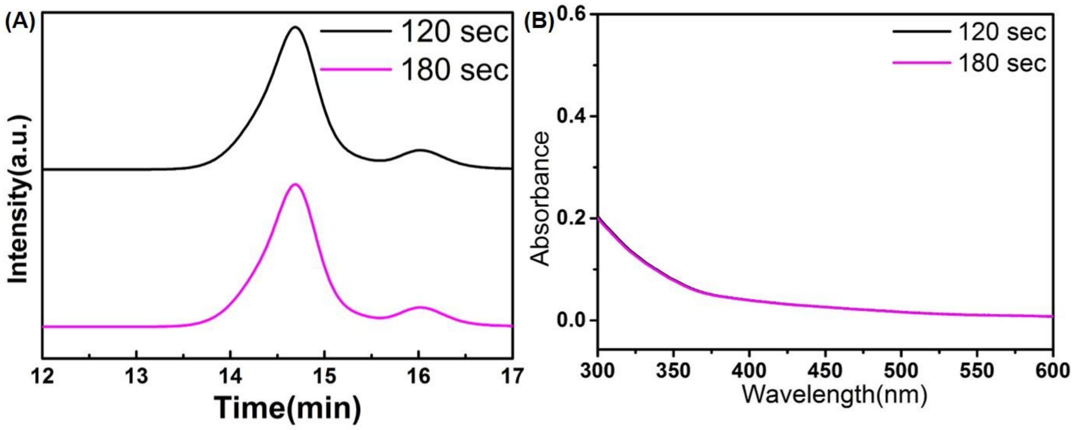

Supplementary Figure 11. (**A**) SEC trace and (**B**) UV absorbance of PS(*hv*-PS')-*b*-PMMA after two different UV irradiation times (120 and 180 s) at 230 ^{o}C.

To confirm whether there were no further cleavage of PS(*hv*-PS')-*b*-PMMA after irradiation for 120 s, we prepared another sample irradiated by UV for 180 s at 230 ^{o}C. Supplementary Fig. 11 gives SEC trace and UV absorbance after two different UV irradiation times (120 and 180 s). From these results, PS(*hv*-PS')-*b*-PMMA completely cleavages under UV irradiation of 120 s at 230°C.

Morphology of PS-Alkyne-*b*-PMMA

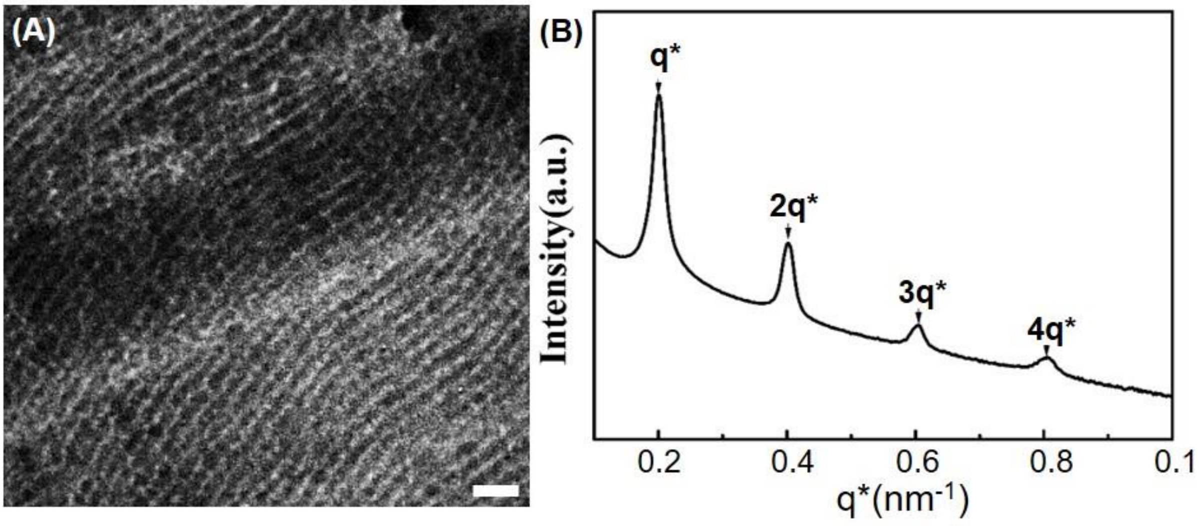

Supplementary Figure 12. (**A**)TEM image and (**B**)SAXS profile of PS-Alkyne-*b*-PMMA. Scale bar in TEM image is 100 nm.

Supplementary Figs. 12A,B give transmission electron microscopy (TEM) image and small angle X-ray scattering (SAXS) profile for PS-Alkyne-*b*-PMMA, respectively. Lamellar microdomains are clearly seen in TEM image. Also, due to SAXS peaks at q*: 2q*: 3q*: 4q*, PS-Alkyne-*b*-PMMA exhibits lamellar microdomains. The lamellar domain spacing (L_0) is 31.3 nm, which is obtained by 2π/q* (q* = 0.201 nm^{-1}).

Thin film morphology with two different thicknesses

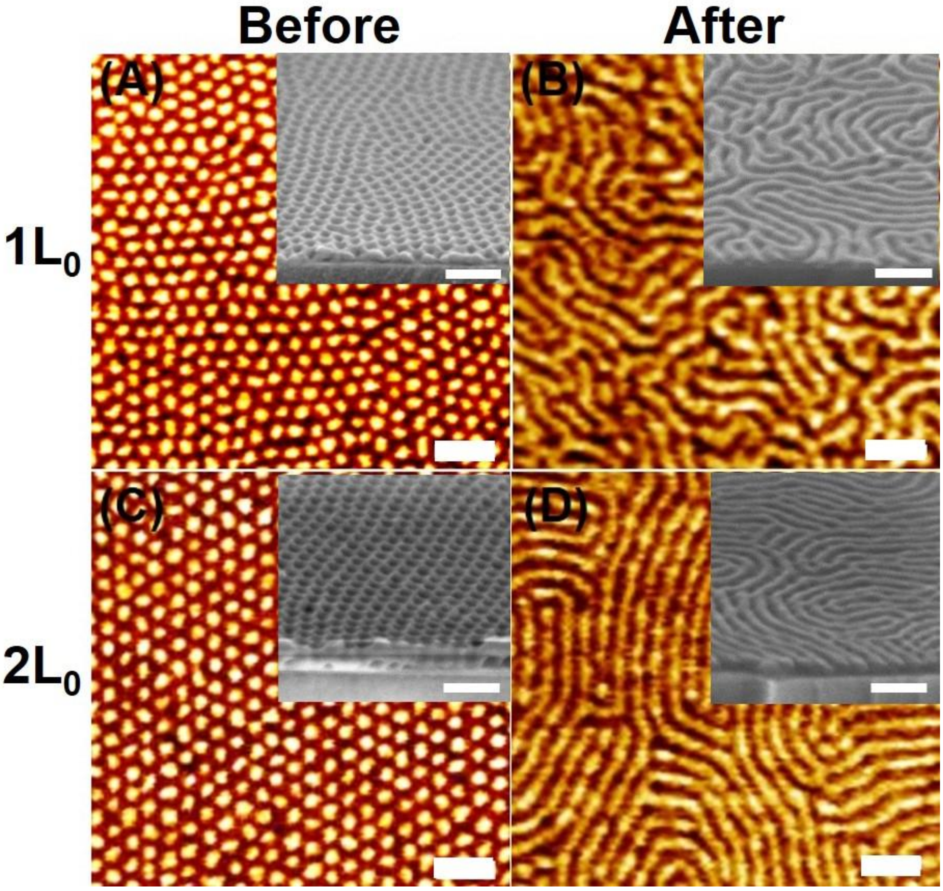

Supplementary Figure 13. Phase contrast AFM and cross-sectional SEM images (inset of each Figure) of thin films with two two thickneesss (**A, C**) before and (**B, D**) after UV irradiation at 230 ^{o}C for 120 s. Top and bottom panels represent film thickness with 28 nm (~ $1L_0$) and 57 nm (~$2L_0$), respectively. Scale bar is 100 nm.

Supplmentray Fig. 13 shows phase contrast atomic force microscopy (AFM) and cross-sectional scanning electron microscopy (SEM) images (inset of each figure) of thin films with two thicknesses (28 nm (~ $1L_0$) and 57nm (~$2L_0$)) before and after UV irradiation at 230 ^{o}C for 120 s. Both samples clearly show dual nanopatterns consistng of dots and lines.

Quantitative analysis of photo-cleavage versus light dose (irradiation time)

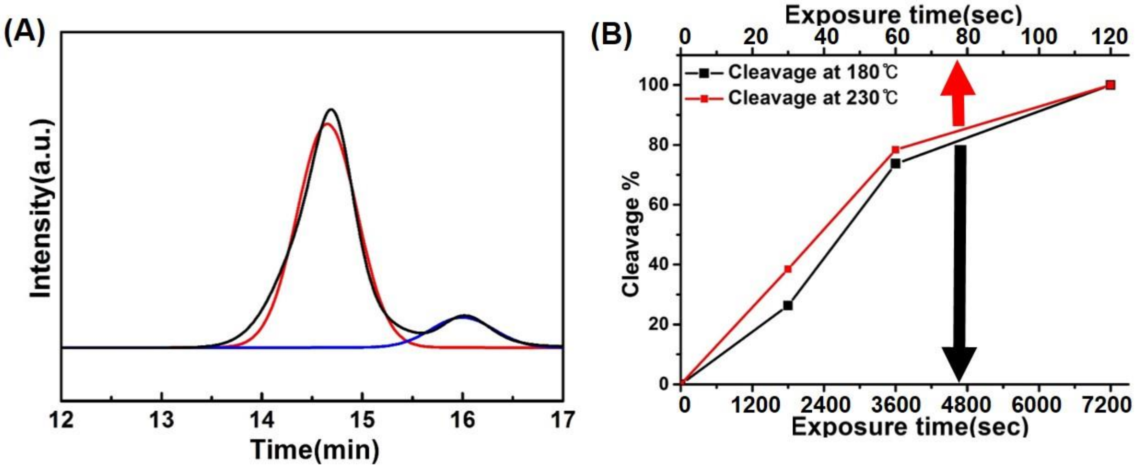

Supplementary Figure 14. (**A**) Peak deconvolution of SEC trace after UV irradiation for 120 s at 230ºC to calculate the amount of PS-*b*-PMMA (red) and homo PS' (blue). (**B**) The degree of the cleavage with UV irradiation time at two temperatures (180 and 230 ºC).

Supplementary Fig. 14A shows deconvolution of SEC trace after UV irradiation (~ 1.3 mW cm^{-2}) for 120 s at 230 ºC to calculate the amount of PS-*b*-PMMA and homo PS'. The smaller and larger peaks correspond to PS-*b*-PMMA and homo PS'. Since the molecular weight of PS-*b*-PMMA and PS' are 46,000 and 11,000 g mol^{-1}, the ratio of the de-convoluted area should be 11,000/(46,000 + 11,000) = 0.19 after perfect cleavage. The degree of the cleavage at a given time was obtained from the ratio of two peaks divided by 0.19.

Considering that the energy of the light source measured by the lux meter was ~ 1.3 mW cm^{-2}, the photo-cleavable linker was broken under lower UV dose (~ 0.156 J cm^{-2}) at 230 ºC. On the other hand, when the sample was irradiated at 180 ºC, higher UV dose (~ 9.36 J cm^{-2}) was required for the perfect cleavage. The degree of the cleavage depended highly on UV irradiation temperature (Supplementary Fig. 14B).

Phase transition depending on UV irradiation temperature

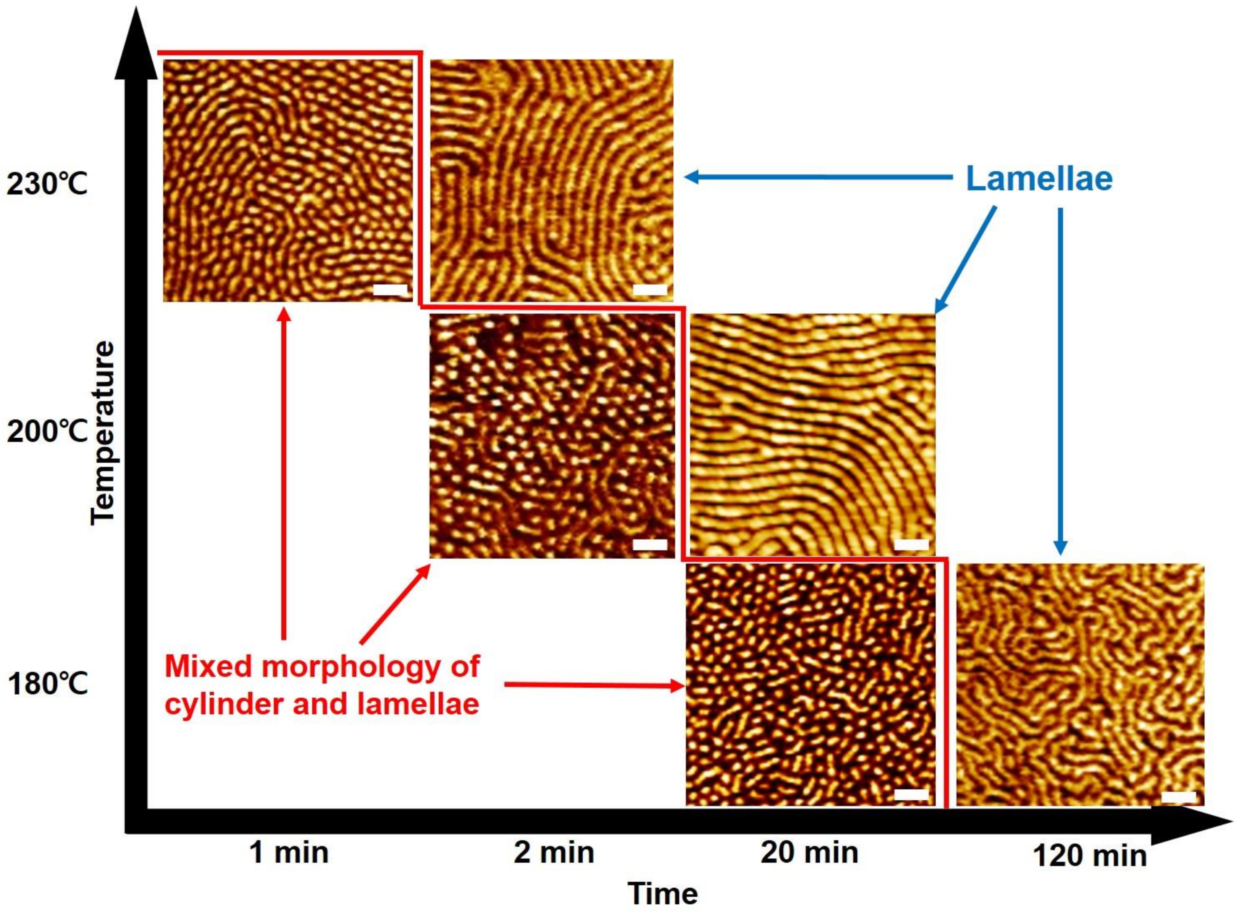

Supplementary Figure 15. Phase contrast AFM images at various UV irradiation conditions. Scale bar is 100 nm.

Supplmentray Fig. 15 shows phase contrast AFM images at various UV irradiation conditions. It is seen that the phase transition from hexagonally packed cyliners to lamellae depends highly on UV irradiation temperatures. An completele transition occurred within 120 s at 230 ^{o}C. With decreasing temperature, this time increased (20 min at 200 ^{o}C and 120 min at 180 ^{o}C). The fast phase transition with increasing tempearture is ascribed to the fast mobilites of cleavaged diblock copolymer and homopolymer chains at higher temperatures.

Phase transition of a block copolymer with high χ

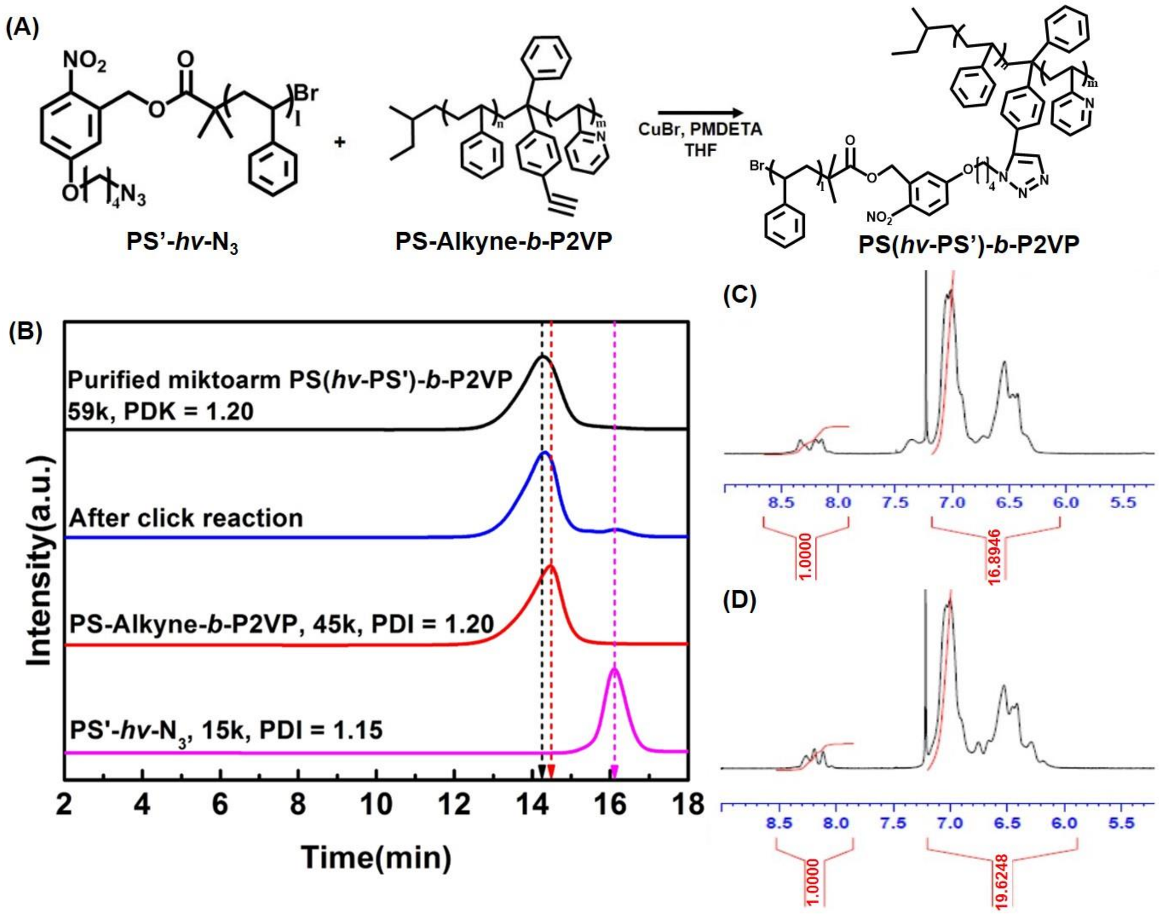

Supplementary Figure 16. (**A**) Synthetic scheme of PS(*hv*-PS')-*b*-P2VP. (**B**) SEC trace of PS-*hv*-N_3, PS-Alkyne-*b*-P2VP, and PS(*hv*-PS')-*b*-P2VP. ^{1}H NMR of (**C**) PS-Alkyne-*b*-P2VP before click reaction and (**D**) PS-(*hv*-PS')-*b*-P2VP after click reaction.

We chose polystyrene-*block*-poly(2-vinyl pyridine) copolymer (PS-*b*-P2VP). The interaction parameter (χ) of PS and P2VP blocks is much larger than that of PS and PMMA, which would also be an advantage for obtaining sub 10 nm pattern for the next generation lithography[5]. We synthesized PS(*hv*-PS')-*b*-P2VP by simply changing monomer from MMA to 2VP using the same synthetic method employed in synthesizing PS(*hv*-PS')-*b*-PMMA, as shown in supplementary Fig. 9A. We characterized the molecular characteristics of PS(*hv*-PS')-*b*-P2VP by SEC and ^{1}H NMR as shown in Supplementary Figs. 16B-D. The molecular weights of PS,

P2VP and PS’ are 32,500, 12,500 and 15,000 g mol^{-1}, respectively, and the volume fraction of (PS + PS’) is 0.80 by using known density of PS (1.05 g cm^{-3}) and P2VP (1.14 g cm^{-3})[6].

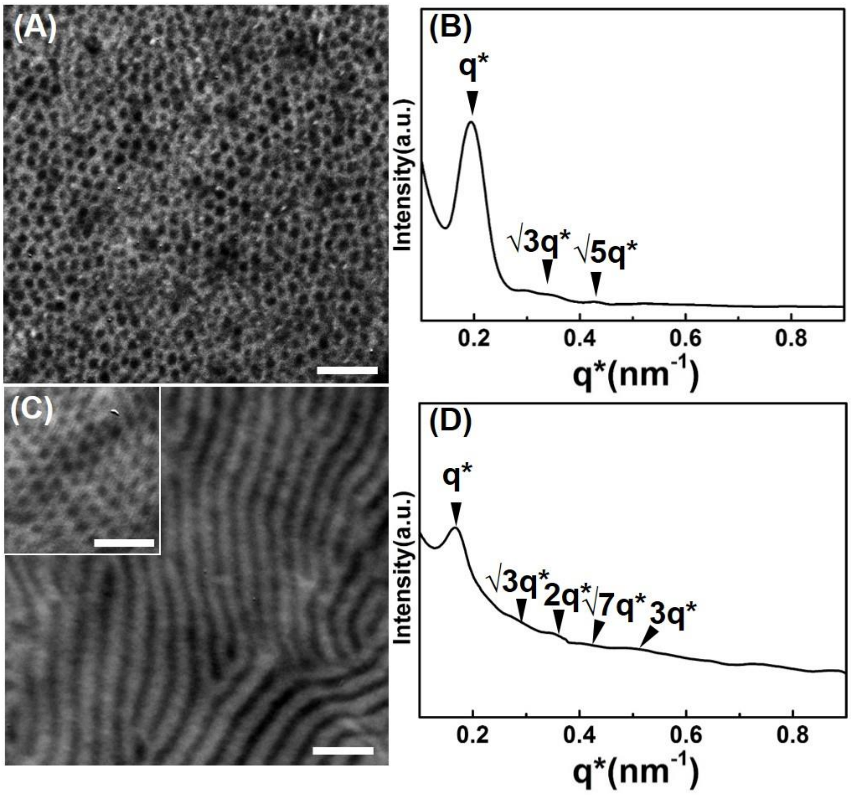

Supplementary Figure 17. (**A, C**) TEM images and (**B, D**) SAXS profiles of PS-(*hv*-PS’)-*b*-P2VP (**A, B**) before and (**B, D**) after irradiation at 230 ^{o}C. The inset of C represents TEM image of a sample cut along the cylindrical axis. The scale bar is 100 nm.

Supplementary Fig. 17 shows TEM images and SAXS profiles before and after UV irradiation at 230 ^{o}C. P2VP microdomains look dark in TEM image because of selective staining by I_2. SAXS profile with peaks at q*:√3 q*:√5q* and TEM image show that neat PS-(*hv*-PS’)-*b*-P2VP exhibits body-centered cubic spherical microdomains, as expected for the volume fraction of PS of 0.8. After the UV irradiation, the mixture of PS-*b*-P2VP and homo PS’ showed hexagonally packed cylindrical microdomains, confirmed by SAXS profiles with peaks at q*:√3q*:2q*:√7q*:3q*. The first peak position (q*) during UV irradiation was

changed from 0.1954 (nm^{-1}) to 0.1673 (nm^{-1}), indicating that the domain spacing (L_0) was increased from 32.2 nm to 37.6 nm. The increased domain spacing was attributed to the nanodomain transformation from spheres to cylinders[7].

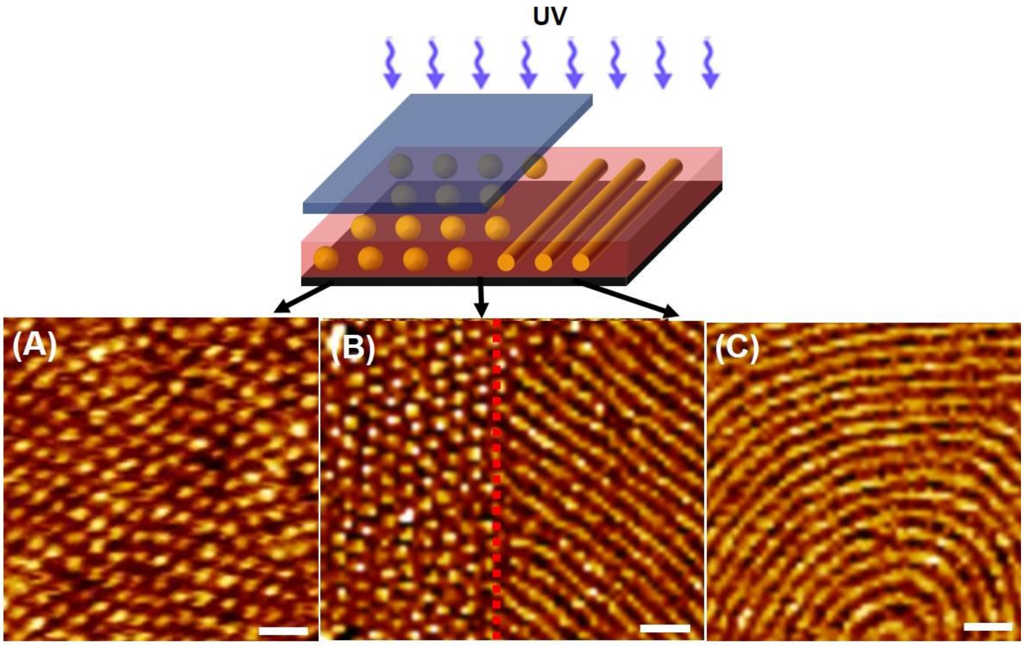

Supplementary Figure 18. Phase contrast AFM images of PS(*hv*-PS')-*b*-P2VP at (**A**) unexposed, (**B**) boundary and (**C**) exposed regions. A boundary marked by dotted line between unexposed (left) and UV exposed (right) region was clearly observed. UV irradiation was performed at 230 ^{o}C for 120 s. The scale bar is 100 nm.

When a thin film with thickness of 50 nm (~ 1.5 L_0) was prepared, we obtained dual nanopatterns of dots and lines resulting from spheres and parallel oriented cylinders, respectively, as given in supplementary Fig. 18. We also clearly see a boundary between spheres and parallel oriented cylinders. Thus, our concept of miktoarm block copolymers with a photo-cleavable linker could be employed for any block copolymer with high χ to fabricate dual nanopatterns at the desired positions.

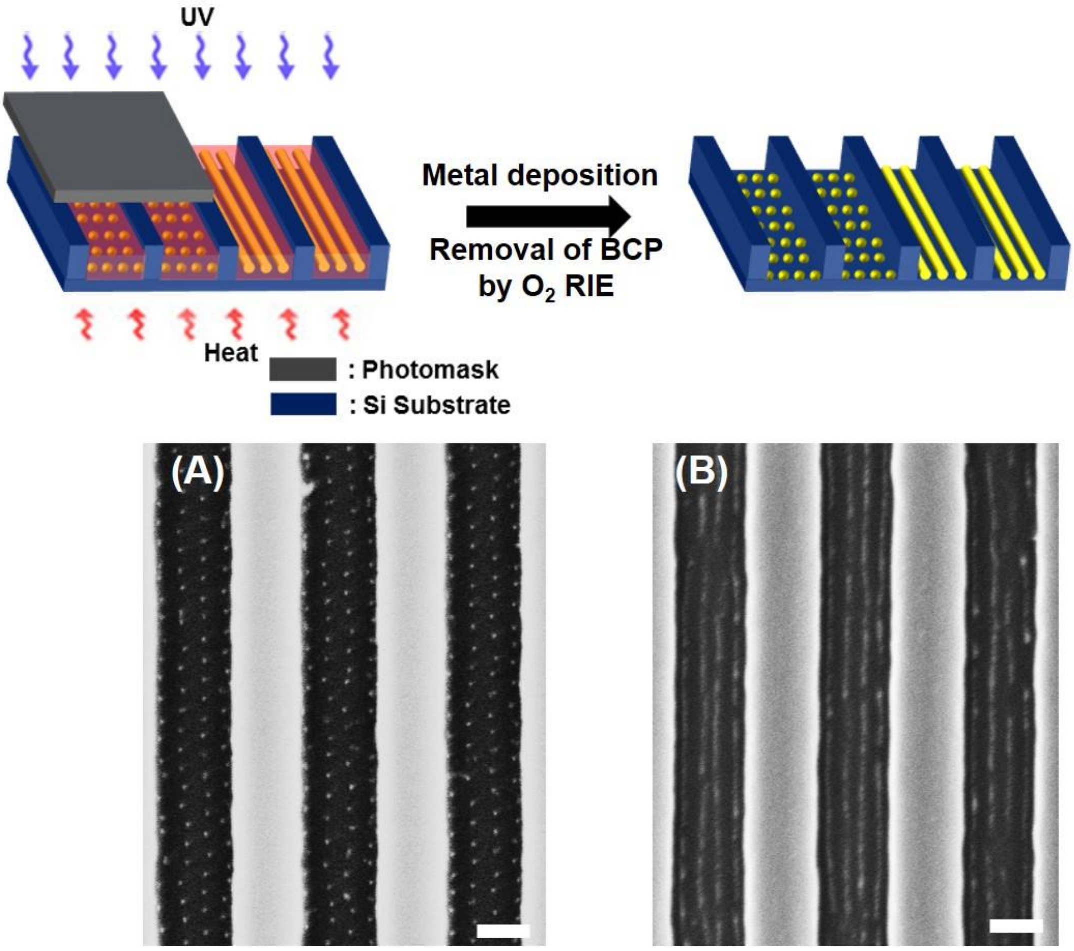

Supplementary Figure 19. Platinum nanopatterns produced from nanopatterns of PS(*hv*-PS')-*b*-P2VP. P2VP spheres in (**A**) unexposed region and parallel oriented P2VP cylinders in (**B**) exposed region were transformed to platinum nanodots and nanowires, respectively. Scale bar is 100nm.

Finally, we obtained a long range ordering of dual nanopatterns of PS(*hv*-PS')-*b*-P2VP nanopatterns by utilizing grapho-epitaxy based DSA with a prepatterned substrate (The width (W) of the trench and the mesa are the same (140 nm) and the thickness of the trench is 70nm). The patterned substrate was fabricated as follows. First, a positive photoresist (K131 produced by DONGJIN Semichem, Korea) was coated on a silicon wafer with 200 nm of oxide layer and baked to remove the residual solvent. Then, it was exposed to 248 nm KrF light source by using a pre-patterned mask. The exposed region of the photoresist was removed by developer

(DPD200 produced by DONGJIN Semichem, Korea) followed by dried at 100 ^{o}C for 1 min. Finally, the photoresist patterns were transferred to silicon oxide layer by capacitively coupled plasma (CCP) dry etching and the residue of the photoresist was completely removed by O_2 plasma. Since PS and P2VP do not have dry (or wet) etching selectivity, it is not easy to see, via SEM images, a long range ordering of P2VP spheres (or P2VP cylindres) after DSA experiment. However, becasuse metal precursors are easily incorporated into only P2VP nanodomain, PS(*hv*-PS')-*b*-P2VP nanopatterns are easily transferred to metal nanopatterns by dipping the block copolymer into a acidic solution containing metal precuross followed by the removal of the block copolymer by O_2 RIE[8,9]. Here, we used platinum precursor solution (10 mL water containing 0.1 mmol of Na_2PtCl_4 and 1 wt% HCl) and O_2 RIE with 250W was performed for 3 min. As shown in Supplementary Fig. 19, we successfully demonstrated a long range ordering of dual nanopatterns consisting of platinum nanodots produced from P2VP spheres at unexposed areas and platinum nanowires obtained from parallely oriented P2VP cylinders. Four line of nanodots and nanowires per trench were generated. This is because the values of W/L_o of spherical and cylindrical nanodomains are 4.3 and 3.7, respectively.

Supplementary References

1. Schlosser M., Schaub B. Instant-ylid: ein lagerfähiges und gebrauchsfertiges Wittig-reagenz. *Chimia* **36**, 396-397 (1982).

2. Hanisch A., Schmalz H., Muller A. H. E. A Modular Route for the Synthesis of ABC Miktoarm Star Terpolymers via a New Alkyne-Substituted Diphenylethylene Derivative. *Macromolecules* **45**, 8300-8309 (2012).

3. Thurn-Albrecht T*., et al.* Ultrahigh-density nanowire arrays grown in self-assembled diblock copolymer templates. *Science* **290**, 2126-2129 (2000).

4. Kaczmarek H., Kaminska A., van Herk A. Photooxidative degradation of poly(alkyl methacrylate)s. *Eur. Polym. J.* **36**, 767-777 (2000).

5. Suh H. S., *et al.* Sub-10-nm patterning via directed self-assembly of block copolymer films with a vapour-phase deposited topcoat. *Nat. Nanotechnol.* **12**, 575-581 (2017).

6. Zha W., *et al.* Origin of the Difference in Order-Disorder Transition Temperature between Polystyrene-block-poly(2-vinylpyridine) and Polystyrene-block-poly(4-vinylpyridine) Copolymers. *Macromolecules* **40**, 2019-2119 (2007).

7. Sakurai S., Kawada H., Hashimoto T. Thermoreversible Morphology Transition between Spherical and Cylindrical Microdomains of Block Copolymers. *Macromolecules* **26**, 5796-5802 (1993).

8. Chai, J.; Wang, D.; Fan, X. N.; Buriak, J. M., Assembly of aligned linear metallic patterns on silicon. *Nat. Nanotechnol.* **2**, 500-506 (2007).

9. Chai, J.; Buriak, J. M., Using cylindrical domains of block copolymers to self-assemble and align metallic nanowires. *ACS Nano* **2**, 489-501(2008).
